# Miniaturized V-band circularly polarized leaky-wave antenna with continuous radiation coverage using modified waveguide and metasurface CSRRs

**DOI:** 10.1038/s41598-023-37362-z

**Published:** 2023-06-22

**Authors:** Yalda Torabi, Homayoon Oraizi, Ali Araghi, Mohsen Khalily

**Affiliations:** 1 School of Electrical Engineering, University of Zanjan, Zanjan, Iran; 2 School of Electrical Engineering, Iran University of Science and Technology, Tehran, Iran; 3 Department of Electronic and Electrical Engineering, University College London, London, WC1E 7JE UK; 4 5G and 6G Innovation Centres (5GIC and 6GIC), Institute for Communication Systems (ICS), University of Surrey, Guildford, GU2 7XH UK

**Keywords:** Engineering, Electrical and electronic engineering

## Abstract

A miniaturized V-band leaky-wave antenna (LWA) with circular polarization and backward-broadside-forward radiation based on a modified half-mode substrate integrated waveguide (M-HMSIW) is presented. The proposed M-HMSIW structure employs broadside coupled complementary split ring resonators to replace metallic vias, resulting in low-cost and fully-planar fabrication advantages over conventional HMSIWs. Each unit cell of the proposed LWA consists of an M-HMSIW in combination with two horizontal stubs and a cross-shaped complementary electric LC slot to provide a proper circular polarization with a composite right/left-handed property. Using this structure, the balanced condition can be obtained for the unit cell; hence a continuous backward-to-forward scanning, including broadside, is achieved. As a result, the proposed LWA with a radiator length of only 3.8 *λ*_0_ provides wide-angle beam scanning from − 53° to + 54° over the frequency range of 61.2 GHz to 73.4 GHz, while maintaining an excellent circular polarization between − 25° and 25°. The maximum gain of the LWA is 11.1 dB which is satisfactory, considering its compactness. The antenna’s performance is experimentally verified, and close agreement between the simulations and measurements is observed.

## Introduction

**Figure 1:**
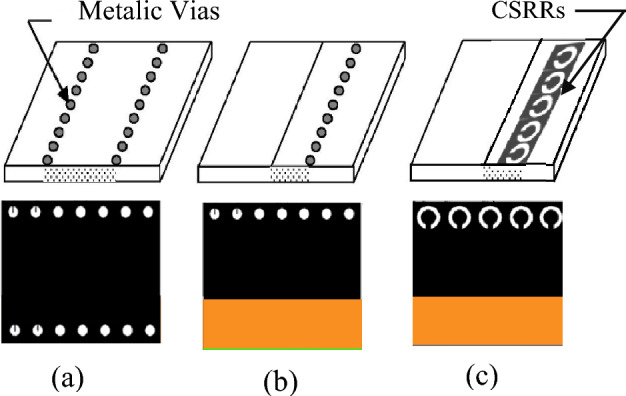
Side and top views of substrate integrated waveguides. (**a**) Conventional SIW, (**b**) conventional HMSIW, (**c**) proposed M-HMSIW based on CSRRs.

With the increasing demand for higher-speed communications and improved system operation, the requirement to move up in frequency is essential. Nowadays, millimeter-wave (mm-wave) communication around the unlicensed 60-GHz frequency band has attracted much attention due to many benefits including higher data rate services, frequency reuse, and strong anti-interference operation^1–4^. This extends to higher bands, especially around the V-band that covers the 57–72 GHz frequency range enabling new applications in the areas of high data-rate communication systems^5^, compact high-resolution imaging radars^4^, 5th generation mobile communications^6^, ultra-high-definition video (UHDV) streaming^7^, remote sensing^8,9^, target detection and tracking^10^, etc. These case scenarios not only require novel technical solutions for market requisites but also demand novel standards for advanced antenna designs. This is due to the considerable increase in losses and fabrication complexity in the mm-Wave frequency range. For example, the IEEE 802.11ad wireless networking standard specifies antenna gain of about 10 dBi with a wide bandwidth of around 14.6% to control massive data traffic, circular polarization to reduce the multipath effect, wide beam scanning range of about ± 50° with a simple feeding network and easy and inexpensive fabrication for bulk production^1^. So far, various high-frequency antenna techniques, like micro-electromechanical systems (MEMS)^11^ and low-temperature co-fired ceramics (LTCCs)^12^, have been approved. However, antennas with the above-mentioned techniques are complex to feed^12,13^, have inadequate scanning ranges^14,15^, suffer from lower gains^11,16^, or possess significant design complexity^12^. Thus, they are not suitable for V-band applications where miniaturization and wide-angle beam scanning are critical. One of the more promising candidates for overcoming these problems is a leaky-wave antenna (LWA), due to its fantastic features such as frequency beam steering capability, high radiating directivity, wide bandwidth, etc.^17,18^. Recently, several V-band LWAs have been reported using various technologies^11,12,19–21^. In^11^, a V-band MEMS-based LWA operating between 52 and 63 GHz was proposed. However, its low gain with a limited scanning range of about 31° makes it unsuitable for V-band applications, where wide-range antenna beam scanning is needed. In^12^, an LTCC substrate integrated image guide (SIIG) LWA was reported within the 60 GHz range (58–67 GHz). However, the multilayered nature of the LTCC makes the overall design complex. In^19^, another LWA with metasurface-loaded Luneburg lens feeding has also been developed, but its performance is unsatisfactory. Reference^20^ describes another novel technique that utilizes dielectric-filled metal grooves to generate a sinusoidally modulated susceptance (SMS) LWA. The V-band LWAs mentioned above suffer from limited scanning ranges with broader beam widths or bulky multilayered structures, requiring considerable design complexity and fabrication costs. On the contrary, substrate-integrated waveguide (SIW), as shown in Fig. 1a, can offer many advantages including low cost, low loss, low profile, and easy integration with other planar circuits over conventional metallic waveguides^22^. As a result, SIW-based LWAs have been widely researched^23–27^. Most of these structures are uniform or quasi-uniform types that work in the fast wave region. Most of these structures are uniform or quasi-uniform types that work in the fast-wave region. Hence, the scanning range of these types of antennas is usually limited to the forward region. In addition, several works have been proposed on half-mode SIW (HMSIW) LWAs, shown in Fig. 1b, as an option for miniaturized applications^28–32^. These structures, half the size of SIW structures can still support all the advantages of SIW structures^33^. Recently composite right/left-handed (CRLH) transmission lines (TLs) have gained attention in LWA design since they enable continuous backward-to-forward beam scanning^34^. To design high-performance LWAs, CRLH media is frequently incorporated into HMSIWs^31,35^. While many CRLH HMSIW LWAs have already been investigated and designed, most provide linearly polarized (LP) radiation or operate at lower frequencies. For V-band LWAs, circularly polarized (CP) waves are preferable to LP waves, as they prevent polarization mismatches and suppress multipath interference^36^. HMSIW LWAs with CP radiation have recently been investigated using different etching techniques^31,37–39^. Nevertheless, most of these LWAs work at lower frequencies, while their design becomes more challenging at higher frequencies (especially V-band) since losses and fabrication complexity increase dramatically. Drilling many holes and metalizing the substrate is a costly and complex process for HMSIWs. The skin effect, proportional to frequency squared, makes metallic losses unavoidable at higher frequencies. Thus, V-band HMSIW LWAs with vertical pin side walls exhibit extreme conductor losses and low radiation efficiency. Further, HMSIW structures are challenging to integrate with active devices since they have metallic vias to the ground, which makes DC bias difficult. Accordingly, designing a V-band fully-planar LWA with CP performance that has an extensive beam steering range, adequate operating bandwidth, high efficiency, and design simplicity is challenging.

This paper introduces a novel V-band fully-planar CP LWA with cross-shaped complementary electric LC (CELC) slots based on a modified HMSIW (M-HMSIW). In contrast to conventional HMSIWs, the proposed M-HMSIW, as shown in Fig. 1c, uses complementary split ring resonators (CSRRs) instead of metallic vias. Compared to conventional HMSIWs, it is fully planar, low-loss, and permits easy fabrication and integration of active components. In the proposed LWA design, each unit cell consists of an M-HMSIW in combination with two horizontal stubs and a cross-shaped complementary electric LC (CELC) slot to provide CP performance with a CRLH property. The full-length LWA with ten matched unit cells acts as a CRLH TL, allowing continuous beam scanning from backward to forward through the broadside. Through dispersion and Bloch impedance simulations, we examine M-HMSIW LWA properties and study how CSRR dimensions influence them. This antenna is optimized using a high-frequency structure simulator (HFSS15) and experimentally verified. The proposed LWA, with a radiator length of only 3.8 λ_0_, can continuously scan from − 53° to + 54° within the 61.2 to 73.4 GHz range while maintaining excellent circular polarization between − 25° and 25°. Broadside radiation occurs at 66.5 GHz with very high CP purity. The LWA has a maximum gain of 11.1 dB, which is reasonable considering its compactness. The main contribution of this work is to incorporate CSRR-based M-HMSIW and slot elements to substantially enhance CP radiation over the V-band frequency range without requiring a cumbersome metallic fabrication process. Finally, compared with most reported CP LWAs in a similar frequency range^28,40^, the developed M-HMSIW CP LWA with cross-shaped CELC slots exhibits higher radiation efficiency and a wider beam-scanning range.

## Proposed structure and operating mechanism

### Antenna configuration

**Figure 2:**
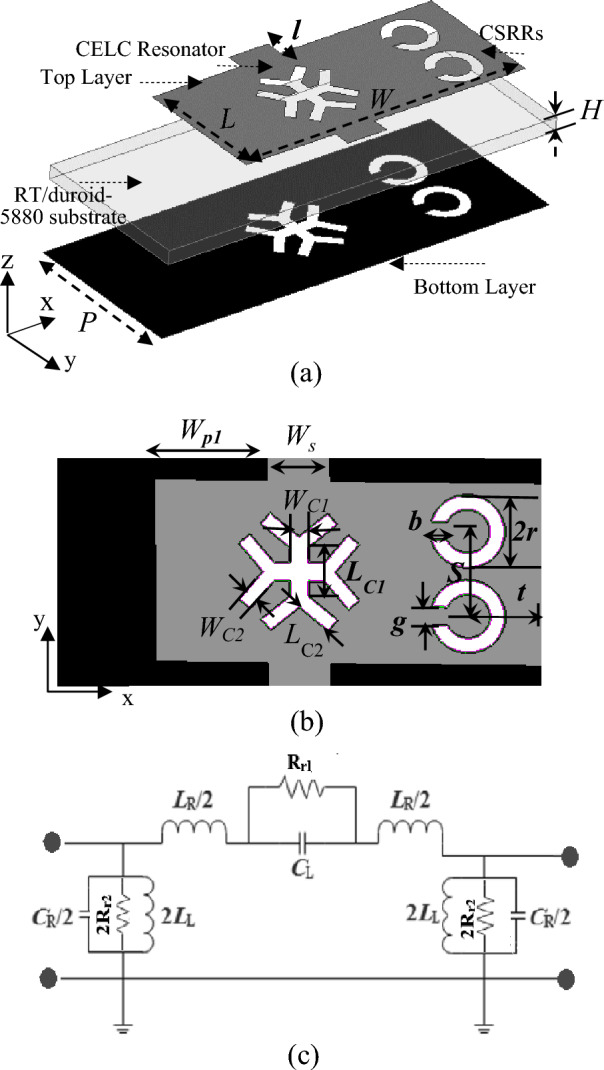
3-D and top view of the proposed CRLH M-HMSIW unit cell structure with the the equivalent circuit model. (**a**) 3-D view with the parameter values of L = 1.5 mm, l = 0.1 mm, W = 3.6 mm, H = 0.254 mm, P = 1.7 mm, (**b**) Top view with the parameter values of W_P1_ = 1.15 mm, W_S_ = 0.5 mm, W_C1_ = 0.15 mm, W_C2_ = 0.1 mm, L_C1_ = 0.4 mm, L_C2_ = 0.3 mm, b = 0.12 mm, r = 0.3 mm, S = 0.7 mm, g = 0.15 mm, t = 0.6 mm and (**c**) The equivalent circuit model of the unit cell.

Figure 2a,b show the 3-D and top views of the proposed LWA unit cell. The unit cell is printed on RT/Durid-5880 with a permittivity of 2.2, loss tangent of 0.0009, and thickness of 0.254 mm. This unit cell consists of an M-HMSIW with broadside CSRRs instead of metalized via holes on the sidewall, two horizontal stubs in the middle of the vertical edges, and a cross-shaped CELC slot at the center.

**Figure 3:**
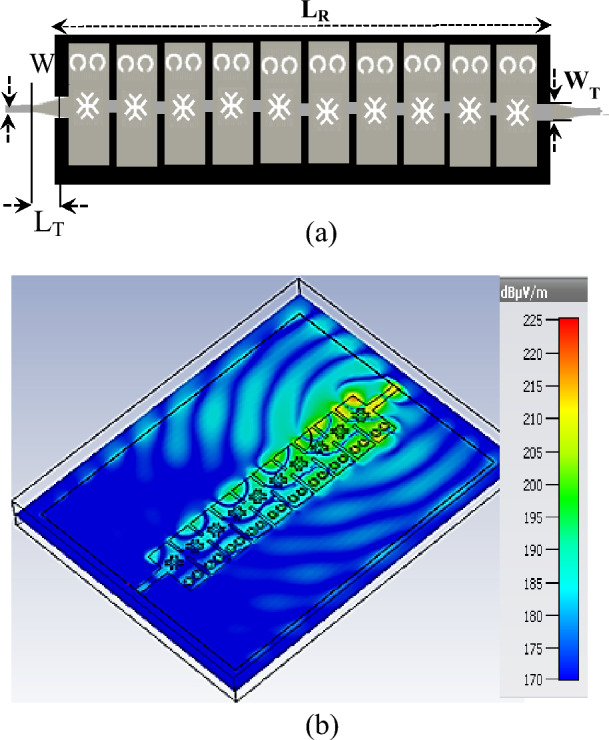
(**a**) Top view of the full-length CRLH LWA with parameter values of L_T_ = 0.25 mm, W_T_ = 0.5 mm, L_R_ = 17 mm, and W_in_ = 0.4 mm, (**b**) Electrical field distribution at 70 GHz.

Figure 2c shows the equivalent circuit model for the proposed LWA unit cell. The unit cell’s top and bottom metal walls can be modeled as series inductance (*L*_*R*_) and shunt capacitance (C_R_) distributed along the structure, which is related to the right-handed (RH) property of the M-HMSIW. Introducing CSRRs on the sidewall, CELC slots, and two-step widths on the top patch of the unit cell changes the distributed capacitance and inductance significantly. A cross-shaped CELC slot and two inter-cell capacitive spacing ensure sufficient series capacitance (C_L_) to achieve a left-handed (LH) structure through the inductive property of the sidewall CSRRs (L_L_). This results in a CRLH M-HMSIW unit cell in addition to its inherent RH property. Furthermore, from the radiation viewpoint, most radiation occurs along the edges of inter-cell gaps and the open side of M-HMSIW, which are represented by (R_r1_) and (R_r2_), respectively. By cascading ten-unit cells with the dimensions indicated in Fig. 2, a full-length CRLH LWA antenna is obtained. Figure 3a shows the top view of the designed LWA with two tapered-line transitions at input and output ports for impedance matching. W_T_ and L_T_ are estimated to match the antenna to 50 Ω. Figure 3b represents the simulated electric field distribution on the top layer of the proposed M-HMSIW LWA at 70 GHz.

The proposed CRLH LWA features backward-to-forward scan capability (including broadside), as described in the following sections. The main beam angle θ of the leaky-wave antenna operating under fast-wave conditions is determined by the ratio between the phase constant β(ω) and the free space wave number* k*_*0*_ as^41^:

1 $$ \theta = \arcsin \left( {\frac{\beta (\omega )}{{k_{0} }}} \right) $$

**Figure 4:**
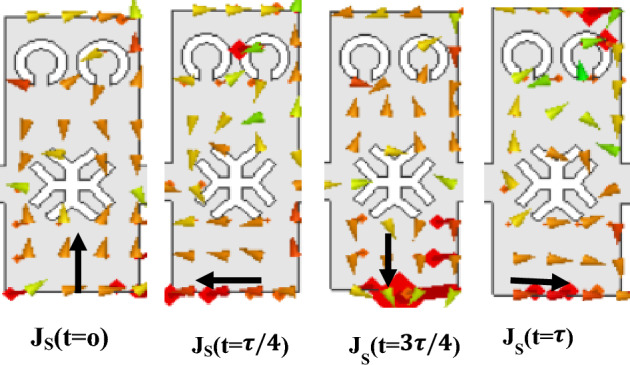
Surface current distribution of the unit-cell at 70 GHz.

It reveals that negative and zero values of *β*(*ω*) lead to negative and zero values of (backward and broadside angles). During wave propagation from the input to the output port, power leaks primarily from the inter-cell gaps on the top metal layer and the open side of M-HMSIW. To produce the desired leakage radiation from the open side, the height-to-width ratio (HWR) of the M-HMSIW structure should be chosen correctly. A higher HWR causes higher back-lobe levels in the E-plane, while a lower value reduces leakage from the open side of the M-HMSIW. Based on the desired cut-off frequency, we estimate the width of the M-HMSIW top layer (W) and then determine the thickness of the substrate (H) to achieve the desired leakage radiation. Due to the radiation from the CELC slots and inter-cell gaps, combined with the M-HMSIW open wall, the radiated electric fields are of almost equal magnitudes, with 90° phase differences^31^. Therefore, the designed antenna might have CP radiation. As a further analysis of the polarization characteristics, we simulate the surface current distribution of the unit cell at 70 GHz. We illustrate the results in Fig. 4. Each section depicts a dominant surface current that rotates counter clockwise, which implies right-handed CP (RHCP) radiation. To properly design the proposed antenna, the M-HMSIW, unit cell, and feed network should be designed accordingly for the particular frequency band of interest used.

### CSRR-based M-HMSIW: characterization and verification of blocking properties

**Figure 5:**
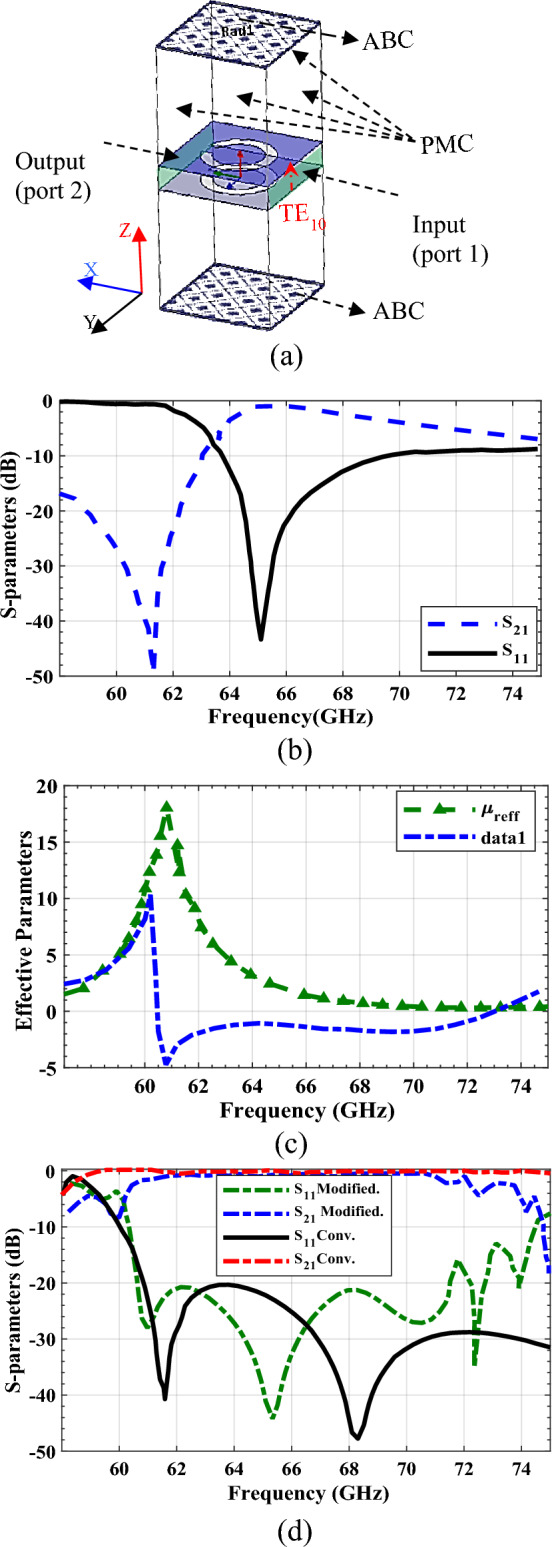
Investigation of broadside-coupled CSRR Structure. (**a**) unit cell geometry and boundary conditions, (**b**) transmission and reflection coefficients vs. frequency, (**c**) real part of effective electric permittivity and magnetic permeability vs. frequency, and (**d**) comparison of the scattering parameters for modified HMSIW utilizing a row of CSRRs in place of via holes as the sidewall and conventional HMSIW.

The first step in designing the CSRR-based M-HMSIW (shown in Fig. 1c), which utilizes broadside-coupled CSRRs instead of metalized via holes, involves determining the properties of its single unit cell. This unit cell consists of two CSRRs etched on the top and bottom metal surfaces, as depicted in Fig. 5a. Due to the coupling between the two complementary rings, the broadside-coupled CSRRs act as an electric dipole^42^, which can be excited by a vertically polarized electric field, blocking in-plane propagation. This electric dipole serves as a building block for the final periodic structure of M-HMSIW, which utilizes double CSRRs instead of each via hole to form one or two rows of CSRRs as the sidewall. By using this approach, the M-HMSIW can achieve similar performance to traditional HMSIW structures while reducing fabrication complexity and cost due to its planarity.

To examine the blocking performance of complementary rings in a unit cell, we employed a 2D periodic metasurface of broadside-coupled CSRRs and conducted a simulation based on the Floquet mode to extract S-parameters. Port boundary conditions are applied to the boundaries marked in green, as shown in Fig. 5a, with a vertically polarized plane wave feeding in port 1 and receiving in port 2. The absorbing boundary conditions (ABCs) are chosen for the top and bottom surfaces of the unit cell (parallel to the x–y plane) and the perfect magnetic conductor (PMC) boundary conditions are imposed on the remaining surfaces to indirectly enforce periodicity, to account for field symmetries along the *x*- and *y*-axes. The analysis of the transmission (S_21_) and reflection (S_11_) scattering parameters for specific geometrical parameters shown in Fig. 2, indicates that the CSRR operates as a resonator in the V-band frequency range (60–70 GHz). The findings are further supported by the minimum transmission region observed around 61 GHz, as illustrated in Fig. 5b. Notably, a high S_11_ level corresponds to low S_21_ values at these frequencies. This implies that the unit cell operates like an electric wall, impeding propagation through the cell.

To further elucidate the wire-like behavior of broadside-coupled CSRRs and prevent in-plane propagation, we employed an effective parameter retrieval method on the CSRR unit cell to investigate its properties. By applying this method, we extracted the constitutive parameters of the effective medium for the broadside-coupled CSRR structure as shown in Fig. 5c. Our analysis revealed that the CSRR structure exhibits a single negative electric permittivity behavior over a broad frequency range of around 10 GHz. This holds great promise for the development of a CSRR-based waveguide with a broad frequency range. Although these results are approximate due to the limited number of CSRRs located along the x- and y-axis and the deviation of the wave interacting with the CSRRs from a TEM wave, they provide valuable insight into the blocking mechanism associated with the broadside CSRRs.

For further insight, we conducted simulations on a 60 GHz M-HMSIW with a waveguide width of 2.45 mm. We incorporated a series of broadside-coupled CSRRs instead of conventional metal vias typically used in HMSIWs. The dimensions of these CSRRs were similar to those shown in Fig. 2. To establish a benchmark for analysis, we also simulated a standard HMSIW with equivalent material properties and length, featuring vias with diameters of d = 0.25 mm and spacing of s = 0.5 mm for a 60 GHz design. The scattering parameters of both waveguides were analyzed and presented in Fig. 5d, demonstrating comparable propagation behavior. Nevertheless, due to the presence of resonant CSRRs, the frequency range of the CSRR-based M-HMSIW was constrained, resulting in a trade-off between bandwidth reduction and fabrication simplicity. However, the remaining operational frequency range provided proper propagation characteristics of approximately 10 GHz, ideal for mm-wave component implementation.

### Design evolution of the CRLH unit Cell based on M-HMSIW for circularly polarization

**Figure 6:**
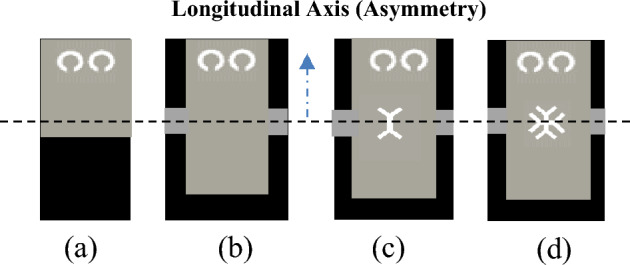
Design evolution of the CRLH unit cell. (**a**) unit cell I, (**b**) unit cell II, (**c**) unit cell III, (**d**) unit cell IV.

Figure 6 illustrates the design evolution of the CRLH unit cell, whose main structure is an M-HMSIW based on broadside coupled CSRRs. The first design iteration, as depicted in Fig. 6a, introduces an M-HMSIW with the via-hole sidewall replaced by a broadside coupled CSRR wall. This results in a planar, cost-effective, and low-loss structure with DC isolation between the top and bottom metal layers when compared to a conventional HMSIW. However, the straight edge of the M-HMSIW acts as a radiator, which cannot produce CP waves with backward-to-forward scanning. To create asymmetry concerning the longitudinal axis and produce elliptical radiation with a controllable axial ratio (AR), a wider M-HMSIW cavity with CSRRs on one side of the unit cell is used in the second design iteration, as shown in Fig. 6b. Continuous beam scanning can also be achieved using CRLH TLs. On the M-HMSIW, the CSRR wall functions as a shunt inductor, allowing the CRLH TL to be designed with only series capacitors. Thus, the second iteration uses a wider M-HMSIW cavity and two horizontal stubs to generate waves in the CRLH radiator by introducing capacitive spacing between adjacent cells. Radiation primarily occurs from inter-cell gaps in the top metal layer and the open side of M-HMSIW, corresponding to series and shunt radiation modes. As discussed in^37^, the series and shunt modes exhibit quadrature phase differences, causing the polarization to be generally elliptical and circular if their radiation contributions are equal. The AR can be controlled by adjusting the amount of asymmetry to the longitudinal axis.

**Figure 7:**
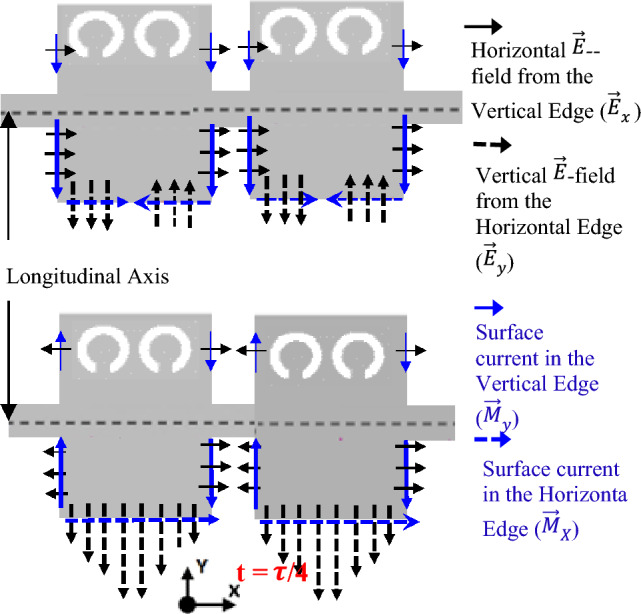
Graphical illustration of the vector field distributions at t = 0 and t = $\uptau$/4 of configuration II.

**Figure 8:**
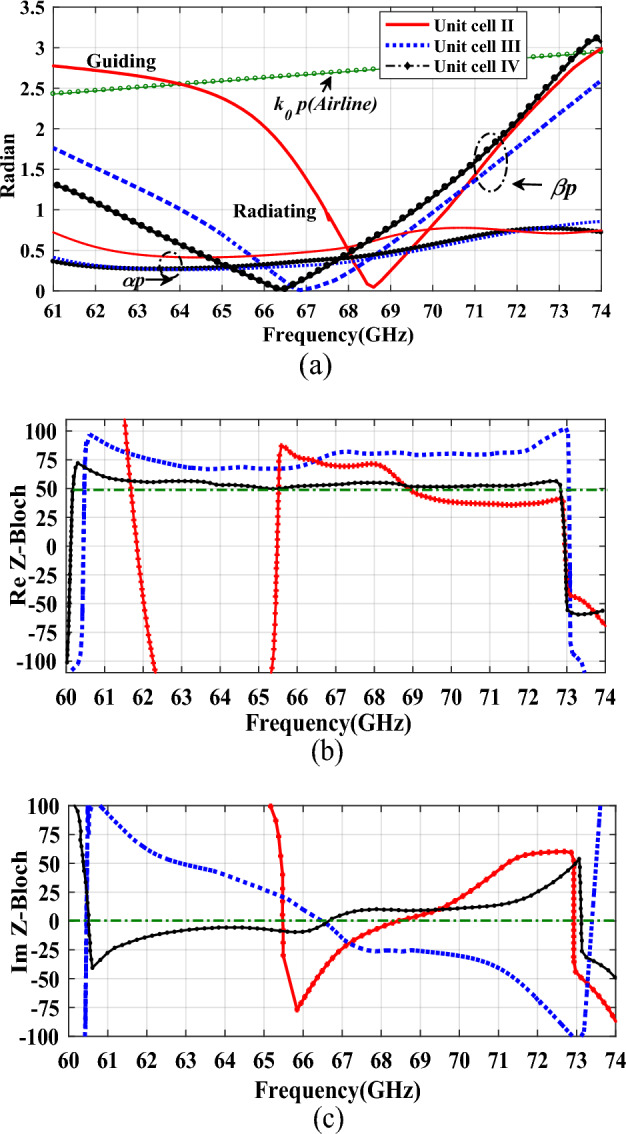
Characteristics of the M-HMSIW unit-cell structures. (**a**) Dispersion diagram, (**b**) Bloch impedance diagram (Real part), (**c**) Bloch impedance diagram (Imaginary part).

To better investigate the polarization performance of the second iteration unit cell, the graphical representation of vector field distributions at t = 0 and t = $\tau$/4 are plotted in Fig. 7. As can be seen, the radiation originates primarily from the horizontal and vertical edges of the top patch. Also, it should be noted that at a zeroth-time instant, the horizontal E fields from the vertical edges of the patch (${\overrightarrow{E}}_{x}$) are dominant, and the fields from the horizontal edge (${\overrightarrow{E}}_{y}$) are canceled. In contrast, at a quadrature time interval, vertical E fields from the horizontal edges of the patch (${\overrightarrow{E}}_{y}$) become dominant, and the fields from the vertical edge (${\overrightarrow{E}}_{x}$) are canceled. Circularly polarized waves can be produced when the two orthogonal electric fields (${\overrightarrow{E}}_{x}$ and ${\overrightarrow{E}}_{y}$) have quadrature phase differences and equal magnitudes. We can achieve this goal by optimizing the dimensions of unit cell II. Despite offering circular polarization with CRLH performance, this radiator has a limited backward scanning range because of its sharp dispersion curve as will be illustrated in the following. A broader backward beam scanning can be achieved by adding transverse I-shaped slots to the patch center, which yields a third iteration of the unit cell (Fig. 6c). The final modification (Fig. 6d, unit cell IV) adds a horizontal I-shaped to the center of the cell to improve impedance matching throughout the entire operating frequency band. We examine the characteristics of the last three M-HMSIW unit-cell structures using dispersion and Bloch impedance diagrams. Unit cell S-parameters can be used to extract dispersion diagrams as^42^:

2 $$ \beta_{n} = \frac{1}{p}\left| {{\text{Im}} \left( {\cosh^{ - 1} \left( {\frac{{1 - S_{11} S_{22} + S_{12} S_{21} }}{{2S_{21} }}} \right)} \right)} \right| $$

3 $$ \alpha = \frac{1}{p}\left| {{\text{Re}} \left( {\cosh^{ - 1} \left( {\frac{{1 - S_{11} S_{22} + S_{12} S_{21} }}{{2S_{21} }}} \right)} \right)} \right| $$

where $\beta_{n}$ stands for the phase constant of the *n*th spatial harmonic, $\alpha$ for the leakage constant related to radiation as a perfect conductor and lossless substrate have been used during simulation, and $p$ for the unit cell length. The S-parameters can be extracted from either the fast-driven-mode simulation or the eigen-mode simulation with the periodic boundary condition. Although the former is less time-consuming, the latter is more accurate^25^. Here, we obtain all S-parameters using Ansys HFSS’s fast-driven-mode simulation. Figure 8a shows extracted dispersion diagrams for the three-unit cell structures. The plotted airline shows that the entire frequency range is divided into guiding and radiating ranges^39^. The broadside frequencies, where the phase constant is zero, separate the backward (LH) and forward (RH) radiating regions in the low and high-frequency bands, respectively. As observed in this figure, the broadside frequency is mainly reduced by introducing the etched transversal I-shape slot and further decreased by adding the horizontal I-shape slot. A transversal I-shaped slot also reduces the dispersion curve slope in the LH frequency band. This results in a broader LH radiation bandwidth with consistent gain and, thus, a wider range of backward scanning, as previously mentioned. All three-unit cell structures show continuous transitions at β = 0, indicating that they have no open stop-bands (OSBs) within the operating bandwidth; hence broadside radiation can be excited there. We also study impedance matching of the unit cells based on Bloch impedance diagrams. Real and imaginary parts of the simulated Bloch impedances are depicted in Fig. 8b,c, respectively, for the three unit cells. The diagrams are derived from the S-parameters of the unit cells as follows^43^:

4 $$ Z_{B} = \frac{{2jZ_{0} S_{21} \sin (\beta p)}}{{(1 - S_{11} )(1 - S_{22} ) - S_{21} S_{12} }} $$

where Z_0_ as a reference impedance for 2-port S-parameters is defined 50 Ω. To ensure proper impedance matching between the microstrip feed line and the unit cell, the real and imaginary parts of the Bloch impedance are designed for 50 Ω and 0 Ω, respectively. Although achieving perfect impedance matching over the entire operating frequency range is challenging, we consider deviations of up to 20 Ω acceptable.

Figure 8b,c show that unit-cell II exhibits poor impedance matching at low frequencies up to around 65 GHz, where leaky modes become radiative. However, it is fairly well matched above that frequency. In addition, the Bloch impedance at the broadside frequency (68.5 GHz) is close to 50 Ω real and zero Ω imaginary, respectively, resulting in nearly perfect impedance matching. Unit cell III with a transverse I-shaped slot exhibits proper impedance matching over the entire frequency range but slightly worse performance above 67 GHz. A horizontal I-shaped slot in unit cell IV achieves excellent impedance matching throughout the entire frequency band between 61 and 73 GHz. In this frequency range, the Bloch impedance is approximately around 50 Ω real and zero Ω imaginary, providing excellent matching.

## Measured results and discussions

### Simulations and experimental specifications

**Figure 9:**
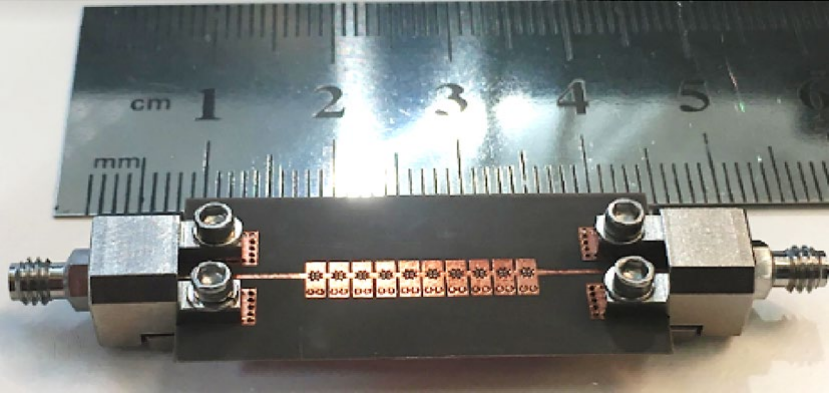
Top view of the fabricated circularly polarized M-HMSIW LWA.

**Figure 10:**
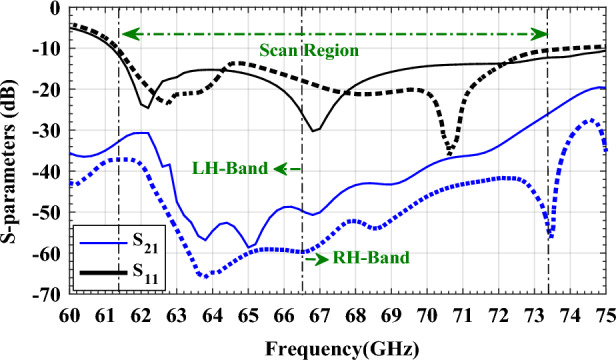
Full-wave simulated (solid) and measured (dashed) S-Parameters versus frequency for the proposed antenna.

**Figure 11:**
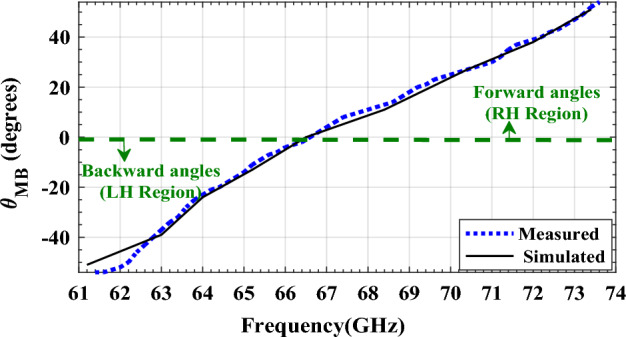
Main beam scanning (θ_MB_) versus frequency of the antenna.

**Figure 12:**
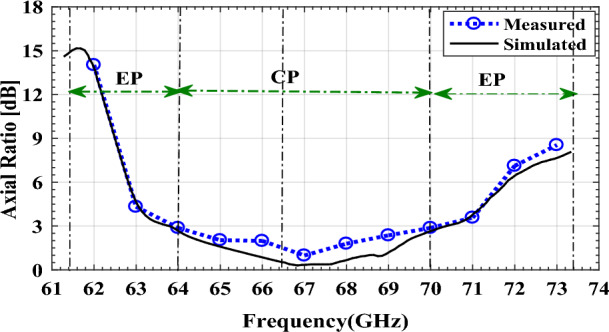
Axial ratio versus frequency at the main beams of the proposed CP LWA (θ_MB_).

**Figure 13:**
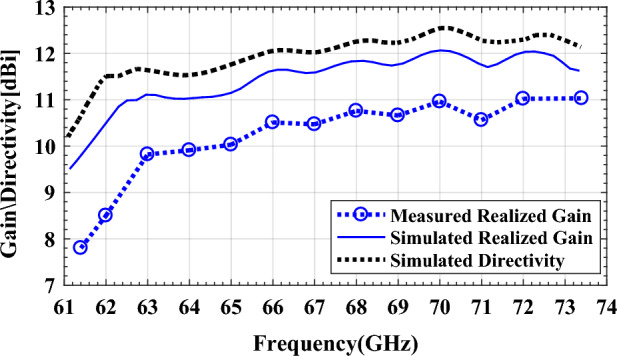
Measured and simulated realized gain and directivity for the proposed antenna.

**Figure 14:**
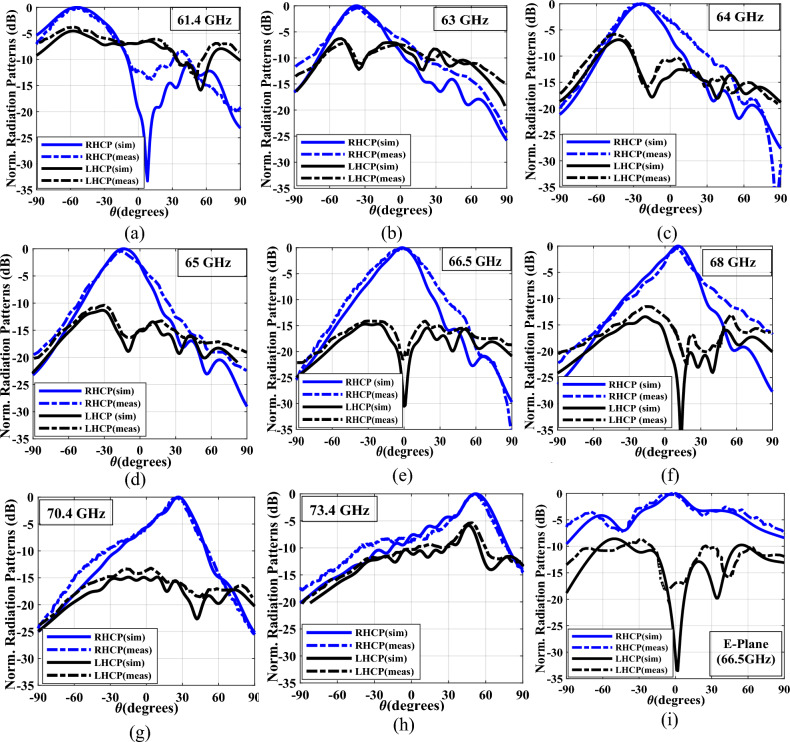
(**a**)**–**(**h**) H-plane co-polarized (RHCP) and cross-polarized (LHCP) normalized radiation patterns of the proposed antenna at different frequencies, (**i**) E-plane pattern at broadside frequency of 66.5 GHz.

**Figure 15:**
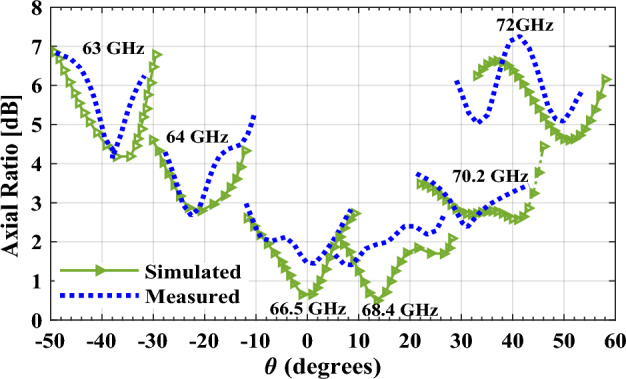
Full-wave simulated and measured axial ratio of the proposed antenna in the principal scan plane (yz plane, φ = 90°) for different frequencies.

For design verification, a prototype of the LWA with ten matched unit cells was fabricated and experimentally characterized as shown in Fig. 9. Using an Agilent N5247A network analyzer, the S-parameters of the fabricated LWA are measured and compared with those of the simulated LWA in Fig. 10. The measured $\left|{S}_{21}\right|$ is somewhat lower than the simulation result, possibly because of fabrication errors and extra losses introduced by connector cables, transition sections, and adaptors. As well known, leaky structures with low $\left|{S}_{11}\right|$ and $\left|{S}_{21}\right|$ and low internal losses (dielectric and conductor losses) ensure adequate radiation levels through slots. Here, the simultaneously measured low $\left|{S}_{11}\right|$ (below − 10 dB) and $\left|{S}_{21}\right|$ (below − 25 dB) values within 61.2*–*73.4 GHz indicate that the system ha satisfactory radiation performance with good impedance matching. For far-field measurements, one port is terminated with a 50 Ω load, and the other is used as a feeding port. Figure 11 shows the LWA’s scan behavior over frequency. In agreement with simulation results, measurement results indicate that the main beam continuously scans between − 53° and 54° within 61.2–73.4 GHz without a stop-band at the broadside direction. A vital feature of the proposed LWA is its ability to maintain circular polarization throughout scanning. AR below 3 dB indicates good circular polarization. Figure 12 shows the AR diagram of the antenna at main beam directions (*θ*_*MB*_) as a function of frequency. It is apparently seen that the AR within the 64–70.8 GHz range is sufficiently low (below 3 dB), which indicates a well-designed CP antenna with a 3-dB AR bandwidth of 6.8 GHz. At the broadside frequency (66.5 GHz), AR reaches its minimum value (0.5 dB in the simulation and 1.2 dB in the measurement) and increases as the scan angle moves toward the endfire or backfire of the LWA, leading to elliptical polarization. Figure 13 illustrates the obtained realized gain by integrating mismatch losses and efficiency losses with the power gain of the antenna, as well as the simulated directivity. It is found that the simulated realized gain varies between 9.5 and 12.1 dBi, while the measured gain ranges from 8.5 to 11.1 dBi. In Fig. 14, a comparison between simulated and measured co-polarized (RHCP) and cross-polarized (LHCP) normalized radiation patterns is presented for different frequencies at the H-plane (yz-plane), including backward, broadside, and forward directions. The dashed lines represent the measured data while the solid lines depict the simulated results. As it can be seen, the measurements are in good agreement with the simulations and the beam scans in the H-plane (*yz*-plane) with frequency variation. The maximum measured cross-polarization rejection level is about − 20 dB at broadside and it decays according to the AR values for off-broadside frequencies. Additionally, Fig. 14 provides E-plane (xz-plane) co-polarized (RHCP) and cross-polarized (LHCP) radiation patterns for the antenna at the broadside frequency of 66.5 GHz. It is clear that the antenna generates an E-plane beam that attains a maximum cross-polarization rejection level of greater than − 17 dB upon measurement. However, due to the non-periodicity of the array along the φ = 0 direction, the beamwidth and SLL values are higher compared to those in the H-plane of the array along the φ = 0 direction. Figure 15 displays the axial ratio of the principal scan plane (H-plane) for various frequencies. As the scan angle moves towards the endfire or backfire of the LWA, the circular polarization transforms into an elliptical polarization. However, it remains highly circular within the − 25° to 25° range. The AR curve’s minima at each frequency can be observed in Fig. 15, occurring at angles of maximum radiation, which is optimal.

### Discussion on the proposed antenna losses

The power budget of the manufactured antenna can be determined by referring to Fig. 10. As a wave travels along the full-length LWA, it experiences gradual attenuation due to various factors, including dielectric loss, conductor loss, and radiation or leakage loss. The total power loss (*P*_*Loss*_) of the 2-port LWA antenna, accounting for all the aforementioned losses, can be computed from the S-parameters S_11_ and S_21_ as in (5)^44^.

5 $$ P_{loss} = 1 - \left| {S_{11} } \right|{}^{2} - \left| {S_{21} } \right|{}^{2} $$

**Figure 16:**
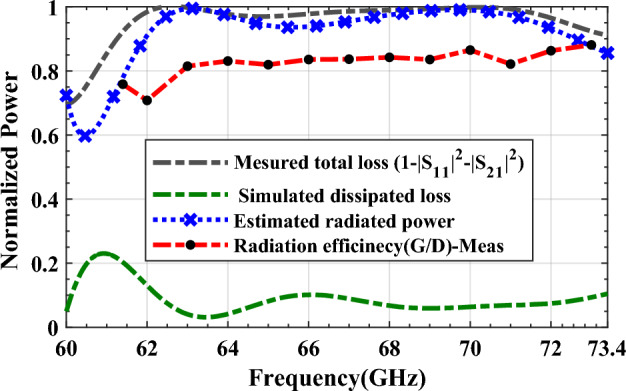
Power budget of the fabricated antenna including dissipated and radiated power with rigorous calculated radiation efficiency defined by gain to directivity ratio (G/d).

Figure 16 illustrates the total power loss, which was determined by (5) in conjunction with the measured S-parameters. To determine the dissipative loss resulting from both dielectric and conductor loss, we conduct a comparative analysis. Specifically, we compare the overall power loss obtained through simulated S-parameters of a lossless structure with that computed from simulated S-parameters of a lossy structure, using the same Eq. (5). The comparison is made possible by running two simulations with appropriate metal and dielectric parameters in the HFSS, utilizing a frequency domain solver. The comparison revealed that less than 10% of input power dissipates throughout the operating frequency range, except in the vicinity of the lower edge of the frequency band where this value reaches up to 24%. This behavior is also illustrated in Fig. 16. Based on the subtraction of simulated dissipative losses from measured total power losses, the radiated power is also estimated to be in excess of 94% for the whole scanning bandwidth (61.4–73.4 GHz), as shown in Fig. 16. Thus, the desired high efficiency has been achieved. However, due to surface waves, efficiency may degrade slightly^44^. For a more precise efficiency calculation, it is necessary to conduct directivity and gain measurements^45^. Directivity requires a comprehensive 3D radiation pattern scan on a spherical grid, which was not feasible with the available instrumentation. However, we measured the gain using a two-antenna setup that involved measuring the antenna under test and a 10 dB standard horn. By this approach, the gain can be measured at different frequencies in the operating frequency range for the proposed LWA. By comparing these values with respective simulated directivity values (see Fig. 13), an estimated efficiency is obtained and depicted in Fig. 16. This result is consistent with the power loss obtained by the earlier method. The leakage (attenuation) constant of the leaky structure can be defined by the S-parameters in (6) or by ABCD parameters in (7)^39,46^.

6 $$ \alpha = \frac{ - 1}{{2L}}Ln\left( {\frac{{\left| {S_{21} } \right|^{2} }}{{1 - \left| {S_{11} } \right|^{2} }}} \right) $$

7 $$ \alpha = \frac{1}{L}(Ln(A) \pm \sqrt {A^{2} - 1} ) $$

The leakage constant as a function of frequency in the LWA concept represents radiation per length. It can usually be used to calculate radiation efficiency as in (8)^39^.

8 $$ R_{e} = 1 - \frac{P(L)}{{P(0)}} = [1 - \exp ( - 2\alpha L)] $$

where $P\left(0\right)$ is the input power at the first port and $P\left(L\right)$ is the remaining power at the end of the leaky structure. Note that the attenuation constant or total leakage constant ($\alpha_{total}$) generally is the sum of all the TL losses per length, which, for a loss-less TL, can be treated as the radiation or leakage constant^46^. This can be stated by (9), where $\alpha_{c}$ and $\alpha_{d}$ are attenuation constants due to conductive and dielectric losses, respectively; and $\alpha_{r}$ is the real radiation or leakage constant.

9 $$ \alpha_{total} = \alpha_{r} + \alpha_{d} + \alpha_{c} $$

**Figure 17:**
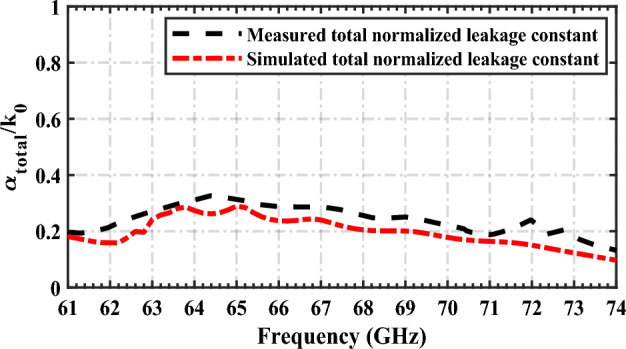
Comparison of simulated and measured total leakage loss of the proposed antenna.

The total normalized leakage constant or total leakage rate ($\alpha_{total} /k_{0}$) curves; where k_0_ is the free space wave number obtained from measured as simulated S-parameters using the formula in (6) are shown in Fig. 17. Both predicted and measured leakage constants agree very well.

### Effect of CSRR wall dimensions on the LWA performance

**Figure 18:**
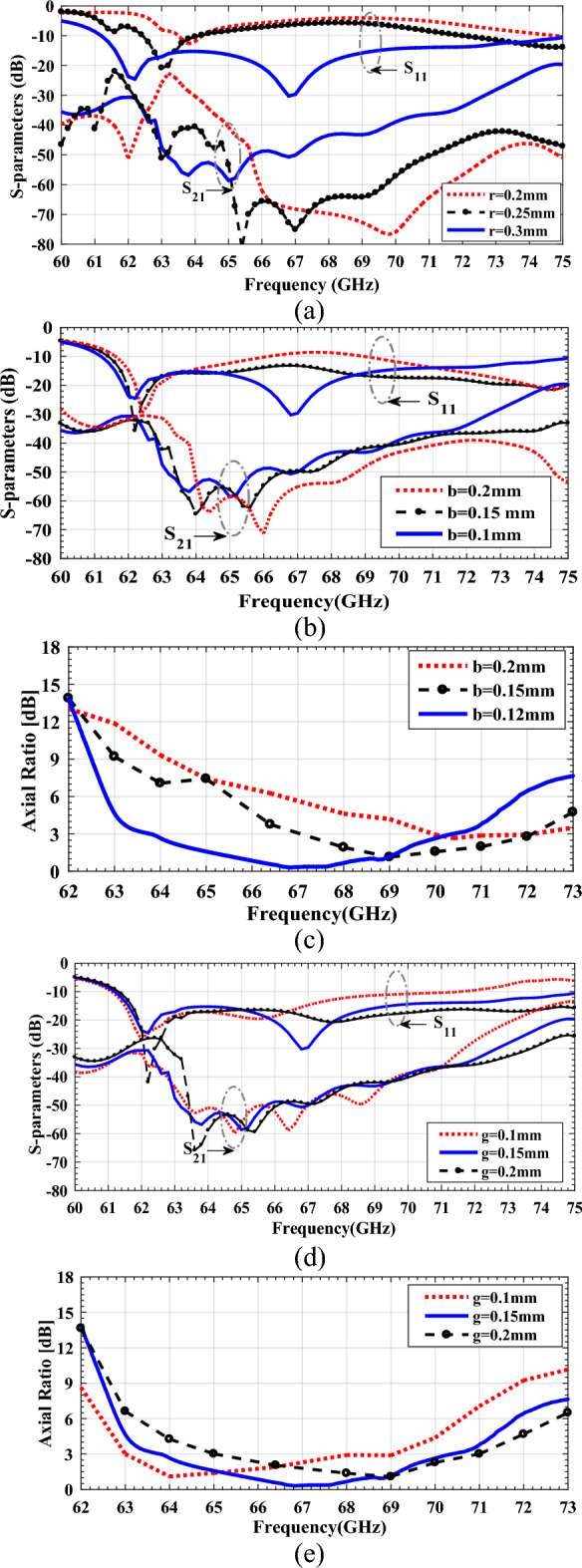
Simulated S-parameters and axial ratio for the proposed LWA for varying CSRR’s dimensions (**a**) S-parameters for varying CSRR’s outer radii, r, (**b**) S-parameters and (**c**) axial ratios for varying CSRR’s ring widths, b, (**d**) S-parameters and (**e**) axial ratios for varying CSRR’s gaps (**g**).

The CSRR wall, which achieves wire-like behavior in a low-loss and fully planar solution, can be regarded as a parallel LC circuit loaded on the side of a microwave device. This LC circuit resonates at a specific frequency, around which band is the CSRR outer radius, *r*, and of someway less importance are the ring width, *b*, and gap, *g*. For instance, operating at a 60–70 GHz frequency band limits the outer radius to about 0.30 mm, whereas there is the freedom to choose a gap or ring width of 0.1–0.2 mm. Figure 18 shows how CSRR’s outer radius, *r*, ring width, *b*, and gap, *g*, influence the matching and polarization performance of the LWA at a substrate height of 0.254 mm and relative permittivity of 2.2. We can estimate the geometric features required for desired circular polarization quality and impedance matching based on these results. As the value of the studied parameter is varied, the values for the other parameters remain identical. Figure 18a illustrates the S-parameters of a full-length LWA for various outer radius configurations. It can be seen that when *r* = 0.3 mm, good impedance matching below − 15 dB is achieved within the majority of the operating bandwidth from 61.2 to 73.4 GHz. However, when r decreases to 0.25 mm, the $\left|{S}_{11}\right|$ values become worse and exceed − 10 dB in both lower and higher frequency bands from 60 GHz to 62.5 GHz and 63.6 GHz to 73 GHz. With a further decrease in *r* to 0.2 mm, the impedance matching performance again deteriorates, and $\left|{S}_{11}\right|$ values remain higher than − 10 dB. As a result, the outer radius of CSRRs has a significant impact on antenna impedance matching. Indeed, a CSRR resonator has a wire-like behavior for frequencies between 60 and 73 GHz if *r* = 0.3 mm. However, for other values of r, CSRR is ineffective, resulting in undesirable behavior. The insertion loss, S_21_, is also sensitive to the outer radius variation of the CSRRs, however, for all values of r, it is seen that the $\left|{S}_{21}\right|$ values remain below − 20 dB. For different ring width values, *b*, Fig. 18b,c show S-parameters and AR values, respectively, as a function of the source frequency. CSRR ring width, *b*, appears to impact OSB and impedance matching at high frequencies, significantly (see Fig. 18b). When increasing the ring width of the CSRRs, an OSB appears at about 66.5 GHz. In the case of $\left|{S}_{21}\right|$, a change in ring width does not have a significant effect; there is only a tiny difference at the high-frequency end. Moreover, we find that the AR is very sensitive to changes in the ring width (see Fig. 18c). Good CP performance is obtained for *b* = 0.1 mm. When *b* increases to 0.15 mm, the AR performance becomes worse. With a further increase in *b* to 0.2 mm, the overall CP performance again deteriorates, with AR values exceeding 3 dB over the entire operating band from 60 to 73 GHz. Figure 18d,e illustrate S-parameters and AR values as a function of source frequency at various gaps, *g*, respectively. When *g* = 0.15 mm, good impedance matching and CP performance are achieved at 64–70.8 GHz with AR values less than 3 dB. When g increases to 0.2 mm or decreases to 0.1 mm, $\left|{S}_{11}\right|$ worsens around broadside frequency, 66.5 GHz, while $\left|{S}_{21}\right|$ remains relatively insensitive except at higher frequencies above 71 GHz (see Fig. 18d). Also, according to Fig. 18e, the AR values in the lower frequency band from 64 to 69 GHz worsen as *g* increases to 0.2 mm. Conversely, when g decreases to 0.1 mm, the AR values in the higher frequency band from 65 to 71 GHz deteriorate. Accordingly, *g* = 0.15 mm was chosen to balance the CP performance in lower and higher frequency bands and yield a good impedance match. Based on this analysis, to design tunable LWAs using CSRR walls with variable geometric parameters, OSB must be avoided, as OSB will degrade far-field characteristics such as AR. In addition, some broadband microwave devices with side-loaded CSRRs can influence impedance matching by varying the radius, width, or gap of CSRRs.

## Comparison with previous works

Table 1 compares the proposed antenna with previously reported LWA structures covering similar frequency ranges. In comparison with references^20^,^40^, the antenna system developed in this paper produces circular polarization with significantly reduced radiator size and superior radiation efficiency and beam scanning range. Reference^43^ antenna exhibits comparable CP performance in the frequency range from 26.5 to 40 GHz with a much larger bandwidth and relatively better gain. However, the proposed structure is advantageous in terms of smaller radiator size with a significantly improved radiation efficiency and smaller radiator size. The reported design in reference^28^ produces LP radiation in the similar frequency region of interest with much better gain and a relatively more extensive beam scanning range. Still, the proposed structure is favorable regarding CP performance with a smaller radiator size and a larger impedance bandwidth. Reference^47^ proposed a LWA with CP performance in the comparable millimeter-wave frequency region; however, the responses were unsatisfactory as we can see that our proposed LWA exhibits a much larger impedance bandwidth and scanning range with a more compact geometry. As a result, based on the above comparison table, the proposed radiating structure prototype produces CP radiation with a broader scanning range and higher radiation efficiency in a more compact geometry than previously reported designs.

**Table 1:** Comparison with previously published Cp LWAs.

Ref	LWA type	Freq. BW (GHz)	Scanning range (degree)	Max gain (dBi)	Pol	3-dB CP BW (GHz)	Broadside radiation	Max radiation effi.	Length/λ0
^20^	Bulky IDW with 3D printing	50–75 (40%)	49° (− 9° to + 40°)	14.2	LP	–	Yes	75%	8.33
^40^	PCB based	57–66 (14.63%)	40° (− 28° to + 15°)	17 (sim.)	LP	–	Yes	87% (sim)	16
^43^	CSIW and two M-shaped slots	26.5–40 (40.6%)	53° (− 28° + 25°)	12.5	CP	26.5–40 (40.6%)	Yes	91% (sim)	10
^28^	HMSI, single-layered PCB	55–65 (16.66%)	120° (− 75° + 45°)	14.5	LP	–	Yes	88.2%	5.6
^47^	Dielectric image line	75* − *85 (11.3%)	18° (− 8° + 10°)	12.7	CP	75 − 85 (11.3%)	Yes	Not given	11.1
This work	CSRR-based M-HMSI CP LWA	61.2–73.4 (18.3%)	50° (− 25° to + 25°)	11.1	CP	64–70.8 (10.22%)	Yes	94% (sim)	3.8

## Conclusion

This paper presents and investigates a novel design approach to V-band leaky-wave antennas featuring circular polarization and wide-beam scanning capabilities. CSRR-based M-HMSIWs and cross-shaped CELC slots were used to develop an effective high-performance antenna. In the proposed M-HMSIW, the broadside CSRR wall replaces the via-hole sidewall, resulting in wire-like characteristics in a low-loss and fully planar solution. Simulation and measurement results within 61.2–73.4 GHz are provided, demonstrating a satisfactory agreement and verifying the design’s functionality. The proposed V-band leaky-wave antenna can operate over 61.2–73.4 GHz with the main radiated beam continuously scanning from − 53° to 54° while maintaining an excellent circular polarization between − 25° and 25° and keeping a reasonably compact geometry (3.8 λ_0_). The main feature of the proposed LWA is preserving a circular polarization while scanning from backward to forward. This antenna has a 3 dB AR bandwidth of 6.8 GHz (64 GHz to 70.8 GHz) and a maximum measured gain of 11.1 dBi. Its size, scan capability, and radiation efficiency surpass those of the latest SIW-based circularly polarized LWAs. It provides CP backward-to-forward scanning functionality for V-band applications.

## Data Availability

The data that support the findings of this study are available from the corresponding author upon reasonable request.
